# MicroRNA biomarkers in cerebrospinal fluid and plasma are associated with reduced cognitive decline in Alzheimer's Disease

**DOI:** 10.1002/alz.083785

**Published:** 2025-01-09

**Authors:** Yang Jiao, Yanfei Ding, Mengyue Niu, Ningdi Luo, Min Zhong, Jun Liu, Aonan Zhao, Yuanyuan Li

**Affiliations:** ^1^ Department of Neurology and Institute of Neurology, Ruijin Hospital affiliated to the Shanghai Jiaotong University School of Medicine, Shanghai, Shanghai China

## Abstract

**Background:**

Despite the promising prospects of using microRNAs in Alzheimer's disease (AD) research, several challenges remain unresolved.

**Method:**

Our study analyzed the changes in microRNA expression levels in cerebrospinal fluid (CSF) and plasma samples and their association with concurrent cognitive decline and hippocampal volume changes in patients with AD using the Alzheimer's Disease Neuroimaging Initiative database.

**Result:**

We observed a significant decrease in plasma hsa‐mir‐125b‐5p levels (P=0.018), significant increase in plasma hsa‐mir‐26a‐5p levels (P=0.017), and significant decrease in mir‐185b‐5p levels in CSF (P=0.017). However, these microRNAs showed no significant differences between the control and AD groups across various ATN subtypes. Lower plasma hsa‐mir‐125b‐5p and hsa‐mir‐26a‐5p concentrations and CSF mir‐185b‐5p concentrations were associated with a faster increase in Alzheimer’s Disease Assessment Scale Cognition 13‐item scale scores (P= 0.011, P=0.009, P=0.018, respectively) and a decline in hippocampal volume (P=0.25, P=0.052, P=0.044, respectively), suggesting a potentially detrimental influence on cognitive and memory impairment in AD progression. CSF mir‐185b‐5p concentrations were positive for amyloid deposition (P= 0.0451, r=0.1754) and negative for fibrillar amyloid levels (P= 0.0392, r=‐0.1647), indicating their potential relevance in AD pathology and the development of AD.

**Conclusion:**

These results highlight the role of CSF and plasma microRNA biomarkers in predicting cognitive and clinical decline in patients with AD.

